# Supplementary material for: Identification of photoperiod-induced specific miRNAs in the adrenal glands of Sunite sheep (Ovis aries)
Source: Front Vet Sci. 2022 Jul 22;9:888207. doi: 10.3389/fvets.2022.888207 (PMC9354845; doi:10.3389/fvets.2022.888207)
Supplement: Supplementary file 1 [file Data_Sheet_1.ZIP › Supplementary file/miRDeep2 file.zip!/miRDeep2 file/Know_Structure/SPLP42A1/oar-mir-181a-1.pdf]

miRBase precursor : oar-mir-181a-1
Total read count : 15965
oar-miR-181a read count : 15917
remaining reads : 48

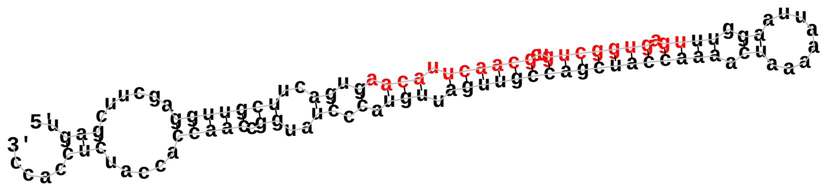

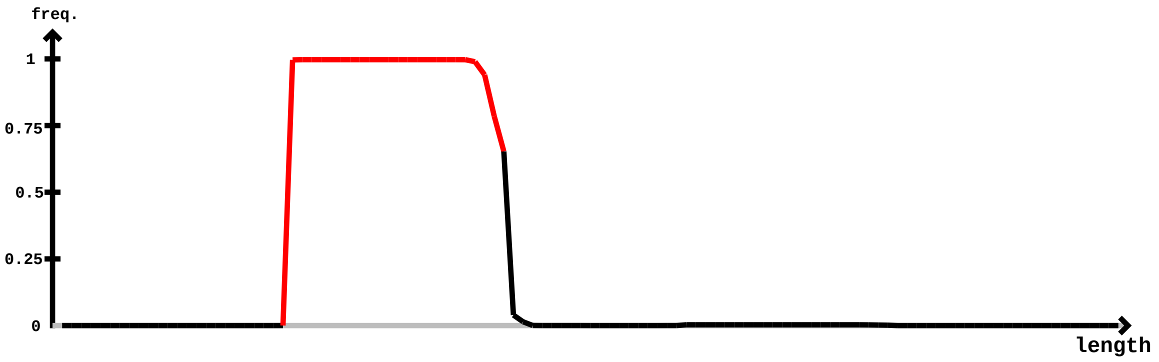


```
                      oar-miR-181a
5'- ugagcuucgagguugcuucagugaacauucaacgcugucggugaguuuggaauuaaaaaucaaaaccaucgaccguugauuguacccuauggccaaccaccaucuccacc   -3'   exp
    .(((......(((((((..((.(.(((.((((((..(((((((.((((.((........)).)))))))))))))))))).))).).))..)).))))).....)))....   reads   mm   sample
    ......................gaacauucaacgcugucgguga..................................................................   1       0    seq
    .......................aacauucaacgcugucgg.....................................................................   2       0    seq
    .......................aacauucaacgcugucggu....................................................................   122     0    seq
    .......................aacauucaacgcugucggug...................................................................   786     0    seq
    .......................aacauucaacgcugucgguga..................................................................   2469    0    seq
    .......................aacauucaacgcugucggugag.................................................................   2112    0    seq
    .......................aacauucaacgcugucggugagu................................................................   9783    0    seq
    .......................aacauucaacgcugucggugaguu...............................................................   403     0    seq
    .......................aacauucaacgcugucggugaguuu..............................................................   219     0    seq
    .......................aacauucaacgcugucggugaguuug.............................................................   6       0    seq
    .......................aacauucaacgcugucggugaguuuggaauu........................................................   1       0    seq
    ........................acauucaacgcugucgguga..................................................................   1       0    seq
    ........................acauucaacgcugucggugagu................................................................   11      0    seq
    ........................acauucaacgcugucggugaguuu..............................................................   2       0    seq
    ..............................................................aaaccaucgaccguugauugu...........................   2       0    seq
    ................................................................accaucgaccguugauugu...........................   4       0    seq
    ................................................................accaucgaccguugauugua..........................   9       0    seq
    ................................................................accaucgaccguugauuguac.........................   10      0    seq
    ................................................................accaucgaccguugauuguacc........................   22      0    seq
```
